# Evaluating ADHD Assessment for Dogs: A Replication Study

**DOI:** 10.3390/ani12070807

**Published:** 2022-03-22

**Authors:** Barbara Csibra, Nóra Bunford, Márta Gácsi

**Affiliations:** 1 Department of Ethology, Institute of Biology, Eötvös Loránd University, Pázmány Péter sétány 1/C, 1117 Budapest, Hungary; marta.gacsi@ttk.elte.hu; 2 Doctoral School of Biology, Institute of Biology, ELTE Eötvös Loránd University, Pázmány Péter sétány 1/C, 1117 Budapest, Hungary; 3 Developmental and Translational Neuroscience Research Group, Institute of Cognitive Neuroscience and Psychology, Research Centre for Natural Sciences, Magyar Tudósok körútja 2, 1117 Budapest, Hungary; nb243610@ohio.edu; 4 MTA-ELTE Comparative Ethology Research Group, Pázmány Péter sétány 1/C, 1117 Budapest, Hungary

**Keywords:** ADHD, inattention, hyperactivity, impulsivity, rating scale, questionnaire, functioning, animal model, domestic dog

## Abstract

**Simple Summary:**

Attention-Deficit/Hyperactivity Disorder (ADHD) is among the most common human neurodevelopmental disorders, characterized by symptoms of inattention, hyperactivity, and impulsivity. Scientists and veterinarians report that—comparably to human children—family dogs can exhibit behaviors similar to symptoms of ADHD. In the present study, we aimed to review the properties of the questionnaire used so far on dogs to test whether it is a reliable tool to assess ADHD-like behaviours, and if it is suitable to detect diagnosable individuals. Similarly to human research, we involved experts (dog trainers) alongside the owners in the evaluation process and compared their ratings. Consistent with earlier results, we could replicate the factor structure of the questionnaire, and item and subscale scores showed high temporal stability. Agreement between owner and trainer ratings were fair (inattention subscale) and moderate (hyperactivity/impulsivity subscale). Three ambiguous items were identified using a version where an ‘I do not know’ answer was also available. Our findings confirmed that the questionnaire is a reliable tool to assess ADHD-like behaviour in dogs. However, to establish whether there are individual dogs diagnosable with ADHD, similarly to human questionnaires, expert ratings and items assessing functional impairment in the daily life of the dogs should be included.

**Abstract:**

The family dog, in its natural environment, exhibits neuropsychological deficits redolent of human psychiatric disorders, including behaviours similar to human Attention-Deficit/Hyperactivity Disorder (ADHD) symptoms. For dogs, Vas and colleagues developed a 13-item questionnaire to measure inattention and hyperactivity/impulsivity (Dog ARS; 2007). We re-assessed, in a large sample of dogs (*N* = 319), psychometric properties of the Dog ARS, to identify possible limitations as a basis for further development. We examined the cross-study stability of factor structure and 40-day temporal stability of item and subscale scores and compared owner-report with expert (dog trainer)-report (*n* = 86), paralleling human parent/teacher assessments. To identify ambiguous items, we administered a modified version (including “I don’t know” options, *N* = 520) to a different sample. We could replicate the factor structure with evidence of good internal consistency and test–retest reliability of both subscales. Agreement between owner and trainer ratings was fair (inattention) and moderate (hyperactivity/impulsivity). Three ambiguous items were identified. Overall, we claim that the Dog ARS is a reliable tool to assess ADHD-like behaviour in dogs, but in its current form, it is not suitable to detect diagnosable individuals, as it does not comprise items assessing functional impairment, and also, the inclusion of owner-expert ratings in the evaluation process would be necessary.

## 1. Introduction

Attention-deficit/hyperactivity disorder (ADHD) is one the most common neurodevelopmental disorder in humans, with worldwide prevalence estimates around 7.5% in children [1] and approximately 3.5% in adults [2,3]. ADHD primarily manifests as inattention, hyperactivity and impulsivity symptoms, though it is a complex and heterogeneous syndrome [4,5,6]. Individuals with ADHD show impairments in a variety of functional domains, including the academic and social domains [7,8].

Beyond rodent models, due to its unique evolutionary past, the family dog is a promising animal model of human socio-cognitive behaviours [9,10,11], and it has been gaining empirical attention as a model for human neurodevelopmental disorders, including autism [12] and ADHD [13]. In the case of ADHD, contrary to rodents, dogs naturally exhibit large ADHD-related phenotypic variability [14,15]. Dogs also show associations between dopaminergic receptor D4 (DRD4) gene and the tyrosine hydroxylase (TH) gene polymorphisms and hyperactivity/impulsivity traits, comparably to human polymorphisms related to ADHD [16]. Finally, dogs exhibit functional impairment in relation to ADHD-like behaviours [17,18], similarly to human children (e.g., [8]).

In humans, evidence-based assessment of ADHD involves a multimethod battery comprising clinical interviews and testing, behavioural observation, and behavioural rating scales completed by relevant informants, such as the child’s parent and/or teacher [19]. Children with ADHD exhibit functional impairments, including in school (e.g., poor achievement) and socially (e.g., family, sibling, and peer relationship problems) [5,20], though behaviours of interest may be cross-situationally different [19,21]. Accordingly, information about the behaviour of the child in different settings is ideally obtained from both parents and teachers is necessary for diagnosis [5,19,21]. Regarding behavioural rating scales for measurement of symptoms and impairment, various questionnaires are available. One such rating scale is the ADHD Rating Scale-IV [22], which measures child behaviour over the past 6 months and consists of 18 items comprising two subscales: inattention (9 items) and hyperactivity/impulsivity (9 items) [22].

Questionnaires originally designed to assess human behaviour have been adapted to measure dog behaviour and also of ADHD-related behaviours and symptoms, including the Dog ADHD Rating Scale (Dog ARS; [14]) and the Dog Impulsivity Assessment Scale (DIAS; [15]). The Dog ARS is an adaptation of the ADHD Rating Scale-IV [22], consisting of 13 items that make up two subscales measuring inattention and hyperactivity/impulsivity. The Dog ARS has been used to assess: breed specificity [23,24,25], comorbidity and prevalence [24,25,26], and genetic correlates [16,27,28,29,30] of ADHD-like behaviours; relations between ADHD-like behaviours and behavioural inhibition [13,31], cognitive bias [31], selective attention [32], and problem solving and temperament [33]; and to compare behavioural observation with owner ratings of inattention and hyperactivity/impulsivity traits [34] and for cross cultural comparisons [35]. In an earlier examination of psychometric properties, Vas and colleagues [14] obtained evidence of acceptable internal consistency for both Dog ARS subscales, but they did not assess test–retest reliability, and to assess evidence of validity, they only examined the relation between age (categories: juvenile, adult and old) and subscale scores. This approach to validity, however, is overly simplistic given the complex and dynamic relations between age and ADHD [36]. In childhood, hyperactive-impulsive symptoms are more dominant than inattention symptoms, but inattention symptoms are more often observed in adolescence and early adulthood [36,37]. Although the manifestation of ADHD symptoms changes with age, the behavioural problems, and functional impairment associated with the disorder (albeit also manifesting differently) persist during development [36,37]. Others evaluated evidence of convergent validity between the Dog ARS and a behavioural test battery probing activity and impulsivity, with results indicating that hyperactivity/impulsivity scores correlated with behavioural performance [16,29].

Lit et al. [38] aimed to replicate the Vas et al. [14] study on a North American dog population, addressing similar research questions and employing a similar design [38]. Lit and colleagues used a slightly modified version of the Dog ARS. One question was removed (“It is excessive, difficult to control, if it lunges it is hard to hold back.”), as the skills assessed could depend on equipment and/or training, and instead of a 4-point, a 5-point Likert response format scale was used (of note, human rating scales also have the 4-point response format to force raters to make a decision on the frequency of symptoms). Consistent with earlier findings [14], age was associated with subscale scores, with older dogs having lower subscale scores, and physical size and sex (when controlling for effects of age) were not associated with subscale scores, suggesting that the Dog ARS has good external validity [38]. The initial factor structure [14] did not replicate, however, as a 3-factor solution (inattention, hyperactivity/impulsivity-1, and hyperactivity/impulsivity-2) fit the data better [38], suggesting a potentially unstable factor structure. Although hyperactivity/impulsivity items loaded onto two factors, these were not hyperactivity and impulsivity—as in humans—but two mixed factors. A design limitation was that a cross-loading item was retained, despite best-practice guidelines recommending elimination of such items [39]. In addition, as previously [14,16,29], test–retest reliability was not examined.

Another measure, the Dog Impulsivity Assessment Scale (DIAS; [15]), albeit not designed to measure ADHD-related behaviours per se, is also relevant as it was developed to measure impulsivity in dogs. The DIAS consists of 18-items that comprise three subscales (see Table 1) [15]. Regarding reliability, internal consistency was unacceptable for responsiveness (α = 0.44). Although they are not the most appropriate measures of test–retest reliability [40,41], Pearson’s correlation coefficients between test and retest scores were calculated and indicated a correlation between scores. In a follow-up study, in which Wright and colleagues’ DIAS results were replicated [42], the responsiveness subscale did not exhibit acceptable agreement across years. Regarding validity, two DIAS items correlated with DIAS total score and subscale scores [15], and this was interpreted by the authors as evincing convergent validity, although item-total correlation is more of an index of internal consistency [43,44]. Further, DIAS total scores correlated with delayed gratification task performance [45], though elsewhere, convergent validity was not supported [46].

For a summary of data on the available questionnaires assessing dog ADHD-related traits, see Table 1 with information on the obtained factor structure and the evaluated aspects of psychometrics across studies.

To contribute to human ADHD research as a model in a reliable and valid manner, finding methods to accurately measure the ADHD characteristics in dogs is crucial. In humans, diagnostic guidelines recommend assessment of symptoms and impairments in different settings (i.e., home and school), obtaining data from multiple informants, usually parents and teachers [21,47,48]. Accordingly, comparability will necessitate canine ADHD research to focus not only on owner but also on expert (e.g., dog trainer) view of dog behaviours, to parallel teacher report.

### Current Study

Our general aim in the current study was to conduct a comprehensive evaluation of the Dog ARS [14] to gather and present evidence on the psychometric properties of—and identify limitations to—the measure, and accordingly suggest potential modifications and refinements that may be implemented. We organized our overarching aim into four specific aims and corresponding research questions as follows.

Aim 1—Compare data obtained in the current and in a prior study on the Dog ARS, to assess the extent to which owners’ evaluation of dog inattention and hyperactivity/impulsivity changed across a 14-year period. 

Q1: Is there a difference between the item ratings of the current (hereafter: C Dog ARS) and the prior sample obtained by Vas and colleagues (hereafter: V Dog ARS)?

Aim 2—Examine the factor structure, test–retest reliability, and external validity of the Dog ARS.

Q1: Is there a difference between the factor structure of the current (C Dog ARS) and the prior sample (V Dog ARS)?

Q2: How reliable is the C Dog ARS across time (40-day test–retest reliability) at the level of the total score, subscale scores, and individual item scores? 

Q3: To what extent are C Dog ARS scores associated with dog age, sex, and training status (external validity)? 

Of note, in examining the factor structure and external validity of the C Dog ARS we simultaneously aimed to determine the extent to which relevant findings obtained earlier [14] are replicable. 

Aim 3—Identification of ambiguous items of the Dog ARS. For this aim, we collected data from owners of a second, separate sample on a modified Dog ARS, where an “I don’t know” (hereafter: IDK) response option was added to all items (hereafter: Dog ARS IDK). Further, for the first time, we also collected data from each dog’s trainer (i.e., experts). 

Q1: Are there any ambiguous items on the Dog ARS IDK for owners (Dog ARS IDK-O), as indicated by a high proportion of owner IDK responses?

Q2: Are there any ambiguous items on the Dog ARS IDK for trainers (Dog ARS IDK-T), as indicated by a high proportion of owner IDK responses?

Aim 4—Compare owner and trainer ratings on the Dog ARS IDK. Especially relative to owners, trainers typically have a large reference group to compare each dog to.

Q1: How reliable is the Dog ARS IDK across raters (interrater reliability), at the level of the total score, subscale scores, and individual item scores?

In Table 2, we summarize our aims and corresponding methods, sample characteristics and sample sizes.

## 2. Materials and Methods

### 2.1. Ethics Statement

The present study was conducted at Eötvös Loránd University in Budapest, Hungary, where animal experiments are overseen by the University Institutional Animal Care and Use Committee (UIACUC). According to the definition of “animal experiments” by the currently operating Hungarian law—the Animal Protection Act—our questionnaire study on dog behaviour was not considered as an animal experiment under the law and was therefore allowed to be conducted without any special permission from the UIACUC. Informed consent was obtained from all dog owners who completed our online questionnaire. Participants were informed about the goals and circumstances of the present study a priori, and they were informed that they may discontinue participating in this research at any time. Participation in the present study was voluntary and anonymous, and the data obtained were used for scientific purposes only. In compliance with relevant data protection laws, personally identifying data were treated confidentially and stored separately from the rest of research data.

### 2.2. Subjects

Participants were recruited through the Department of Ethology participant pool and website, popular social networking sites, and via snowball sampling. Dog trainers were recruited via the dogs’ owners (owners were asked to involve the trainer working with their dog). Sample sizes differed across research questions and were accordingly indicated separately below for each question. Information on sample demographics for the different samples are summarized in Section S3 in the Supplementary Material.

### 2.3. Measures

Questionnaires were completed online (see Sections S1 and S2 in the Supplementary Material) between January 2017 and December 2019 in Hungary. The questionnaires were submitted to the participants in their native language, in Hungarian.

Respondents were asked to provide their own name and e-mail address as well as the dog’s name, breed, date of birth, sex, neuter status, and training status (none-basic-advanced). Dog inattention and hyperactivity/impulsivity were measured using two versions of the Dog-ADHD Rating Scale (see below). 

#### 2.3.1. Dog ARS

Inattention and hyperactivity/impulsivity in dogs were measured using the Dog-ADHD Rating Scale [14], a 13-item (6 inattention items and 7 hyperactivity/impulsivity items) owner-report measure of inattention and hyperactivity/impulsivity in dogs. Owners indicate the frequency with which their dog behaves as described in each item (4-point Likert-type response format, ranging from ‘never’ to ‘very often’). Inattention and hyperactivity/impulsivity subscale scores were calculated by summing item scores for each subject. Greater scores indicate greater difficulties with inattention and hyperactivity/impulsivity. For the questionnaire, see Section S1 in the Supplementary Material.

#### 2.3.2. Dog ARS IDK

The Dog ARS was modified via inclusion of an “I don’t know” (IDK) response option for each question. The questionnaire was used to assess owner (Dog ARS IDK-O) and trainer (Dog ARS IDK-T) ratings of dogs. For the questionnaire, see Section S2 in the Supplementary Material.

#### 2.3.3. Covariates of Non-Interest

Relevant covariates that have been previously hypothesized or shown to be associated with differences in canine inattention and hyperactivity/impulsivity, were dogs’ owner-reported age, sex, and training status [13,14,31].

### 2.4. Methods, Statistical Analyses, and Samples Used for the Different Aims in the Present Study

#### 2.4.1. Aim 1/Question 1: Is There a Difference between the Item Ratings of the Current (C Dog ARS) and the Prior Sample (V Dog ARS)?

Previously obtained data by Vas et al. [14] on *N* = 220 dogs (V Dog ARS), 106 males and 114 females, with *M*age = 35.56 months, *SD* = 7.79 were used for the present question. They reported no data on neutering status. In the current study we used the Dog ARS [14] questionnaire without modification (C Dog ARS). C Dog ARS data were available for *N* = 319 dogs: 162 male (83 intact, 79 neutered) and 157 female (61 intact, 96 spayed) (*M*age = 48.44 months, *SD* = 36.23).

#### 2.4.2. Aim 2/Question 1: Is There a Difference between the Factor Structure of the Current (C Dog ARS) and the Prior Sample (V Dog ARS)?

To address this research question, we used the Dog ARS [14]. The analysis sample was the same as for Aim 1/Question 1, the C Dog ARS and the V Dog ARS.

#### 2.4.3. Aim 2/Question 2: How Reliable Is the C Dog ARS across Time (40-Day Test–Retest Reliability) at the Level of the Total Score, Subscale Scores, and Individual Item Scores?

For assessing test–retest reliability, the C Dog ARS was completed, on average, 40 days apart, with a range of 24 to 49 days. Data for these analyses were available for *n* = 140 dogs: 74 male (37 intact, 37 neutered) and 66 female (27 intact, 39 spayed) dogs (*M*age = 48.65 months, *SD* = 36.58).

#### 2.4.4. Aim 2/Question 3: To What Extent Are C Dog ARS Scores Associated with Dog Age, Sex and Training Status (External Validity)?

For the present research question, we collected data with the Dog ARS [14]. The analysis sample was the same as for Aim 1/Question 1, the C Dog ARS, and the V Dog ARS.

To examine external validity (relations with age, sex, and training status on inattention and hyperactivity/impulsivity) and make valid comparisons across prior [14] and the current findings, training status was indexed as “none” (no training), “basic”, or “advanced” (IPO Schutzhund, rescue, service, or gun dog exam) as in Vas et al. [14]. In that study, age was also treated as a categorical variable (juveniles, adults, and old dogs), and interactions between age, sex, and training status were not examined. In the current study, age was treated as a continuous variable, sex and training status as categorical variables, and interactions were considered. 

#### 2.4.5. Aim 3/Question 1: Are There Any Ambiguous Items on the Dog ARS IDK-O, as Indicated by a High Proportion of Owner “I Don’t Know” Responses?

To identify ambiguous questions, the Dog ARS was modified via inclusion of an IDK response option for each question (Dog ARS IDK). Assuming that respondents select the IDK option when a question is difficult to respond to or they are uncertain in their rating, higher IDK response counts may indicate items that need to be changed in the future.

The analysis sample consisted of *N* = 520 dogs for the Dog ARS IDK-O (independent sample from the *N* = 319 sample, see Table 2). This sample consisted of 227 male (120 intact, 107 neutered) and 293 female (98 intact females, 195 spayed females) dogs (*M*age = 55.01 months, *SD* = 39.59).

#### 2.4.6. Aim 3/Question 2: Are There Any Ambiguous Items on the Dog ARS IDK-T, as Indicated by a High Proportion of Trainer “I Don’t Know” Responses?

The Dog ARS IDK-T (with IDK response option) was used to reveal ambiguous items for trainers. We also included in the analysis the owner ratings (using the Dog ARS IDK-O) which were available for the same dogs that were rated by the trainer.

The analysis sample was a subsample of the sample used for Aim 3/Question 1 (see Table 2). Linked to the previous research question, trainer ratings were available for *n* = 86 dogs: 40 male (19 intact, 21 neutered) and 46 female (13 intact, 33 spayed) dogs (*M*age = 44.31 months, *SD* = 28.96). Fifteen trainers completed the questionnaire, with a range of minimum 1 to maximum 20 responses (dogs) per trainer.

#### 2.4.7. Aim 4/Question 1: How Reliable Is the Dog Ars across Raters (Interrater Reliability), at the Level of the Total Score, Subscale Scores, and Individual Item Scores?

To compare owner and trainer ratings, the Dog ARS IDK-O and the Dog ARS IDK-T were used.

The same data were used for assessing owner–trainer inter-rater agreement as for Aim 3/Question 1 (see Table 2). On average, owners and trainers completed the rating scale 24 days apart.

### 2.5. Statistical Analyses

Statistical analyses were conducted in IBM SPSS Statistics version 22.0.0.0. for Aim 1, Aim 2, and Aim 4. For Aim 3 and Aim 4 IDK response and agreement rate were calculated and presented using Microsoft Excel 2010 version 14.0.7268.5000 (32 bit). Analyses used for the different aims and research questions are described in detail below.

#### 2.5.1. Aim 1/Question 1: Is There a Difference between the Item Ratings of the Current (C Dog ARS) and the Prior Sample (V Dog ARS)?

Independent samples *t*-tests (adjusted for Levene’s test in the case of unequal variances) were conducted to evaluate differences between the C Dog ARS and the V Dog ARS sample means of each item, with *p* < 0.05 considered significant.

#### 2.5.2. Aim 2/Question 1: Is There a Difference between the Factor Structure of the Current (C Dog ARS) and the Prior Sample (V Dog ARS)?

Exploratory factor analysis was used with varimax rotation to explore the factorial structure, and item loadings and findings were compared with those obtained previously [14]. Internal consistency for the inattention and hyperactivity/impulsivity subscales was estimated using Cronbach’s alpha. The independence of the subscales was evaluated with Pearson correlation.

#### 2.5.3. Aim 2/Question 2: How Reliable Is the C Dog ARS across Time (40-Day Test–Retest Reliability) at the Level of the Total Score, Subscale Scores, and Individual Item Scores?

To estimate test–retest reliability, intraclass correlation coefficients (ICC) with corresponding 95% CIs were computed, as they measure agreement and account for both consistency of performances from test to retest, as well as for the systematic change in the mean [41]. ICCs represent the ratio of between-subjects variance to total variance for assessing test–retest reliability when observations are not independent [49]. ICCs can range from −1 to 1 and, in accordance with convention, were interpreted as follows: 0–0.2 as poor, 0.3–0.4 as fair, 0.5–0.6 as moderate, 0.7–0.8 as strong, and >0.8 as almost perfect [50]. Of note, it is possible for ICCs to be negative when the within-group variance exceeds the between-groups variance, suggesting a measure is not reliable. We also reviewed the consistency of owner responses at the item level by calculating an agreement rate (%).

#### 2.5.4. Aim 2/Question 3: To What Extent Are C Dog ARS Scores Associated with Dog Age, Sex, and Training Status (External Validity)?

The association between age, sex, training status, and their interactions and the dependent variables, i.e., inattention and hyperactivity/impulsivity, were examined in generalized linear mixed models with backward elimination. Age was entered as a covariate, sex, and training status as fixed factors and subject as random factor. Following backward elimination, variables were removed in order of decreasing significance, starting with the interactions, until only significant variables were in the model. 

Tweedie with log link option was used as model type, given that the dependent variables had zero scores and skewed distributions, resulting in non-normal distribution of residuals. Sidak correction was applied to account for multiple comparisons. Assumptions were considered prior to all analyses, these were met. 

#### 2.5.5. Aim 3/Question 1: Are There Any Ambiguous Items on the Dog ARS IDK-O, as Indicated by a High Proportion of Owner “I Don’t Know” Responses?

The proportion of IDK responses for each item are calculated (i.e., summed) and presented. For identifying a cut-off point, when an IDK response proportion was considered high, we calculated the average IDK response rate, and then, if the absolute difference between the average and the particular “IDK” response proportion was higher than 150%, we considered those questions as potentially problematic.

#### 2.5.6. Aim 3/Question 2: Are There Any Ambiguous Items on the Dog ARS IDK-T, as Indicated by a High Proportion of Trainer “I Don’t Know” Responses?

The analysis was the same as for Aim 3/Question 1.

#### 2.5.7. Aim 4/Question 1: How Reliable Is the Dog ARS across Raters (Interrater Reliability), at the Level of the Total Score, Subscale Scores, and Individual Item Scores?

Intraclass correlation coefficients (ICC) with corresponding 95% CIs were computed for measuring owner–trainer inter-rater agreement at the level of the total score and subscale scores. To review the consistency of owner–trainer ratings at the item level, an agreement rate (%) was calculated.

## 3. Results

### 3.1. Aim 1/Question 1: Is There a Difference between the Item Ratings of the Current (C Dog ARS) and the Prior Sample Obtained by Vas and Colleagues (V Dog ARS)?

#### Stability of Item Scores

Descriptive statistics for each item are presented in Table 3. Comparing V Dog ARS and C Dog ARS data, inattention items 1, 2, and 12 as well as hyperactivity/impulsivity item 6 differed across the datasets (see Table 3). The mean scores on inattention items 1, 2, and 12 mean were higher in the current sample whereas the mean score on hyperactivity/impulsivity item 6 was higher in V Dog ARS sample (see Table 3).

### 3.2. Aim 2/Question 1: Is There a Difference between the Factor Structure of the C Dog ARS and V Dog ARS?

#### 3.2.1. Reliability of Subscales

The inattention subscale with six items had good internal consistency, α = 0.81. All items appeared retainable, as Cronbach’s alpha would not measurably improve with exclusion of any (Table 3). All items correlated with the total subscale (all *r*s ≥ 0.45) (Table 3).

The hyperactivity/impulsivity subscale with seven questions had lower but acceptable internal consistency, α = 0.78. As with the inattention subscale, there would be very little improvement with exclusion of any item (Table 3) and all individual items correlated with the total subscale (all *r*s ≥ 42).

#### 3.2.2. Factor Analysis

The scree plot indicated two factors (Factor 1 and Factor 2, see Table 4). Item 4 (“My/this dog leaves from its place when it should stay.”) was deleted after the first run of the factor analysis because it loaded >0.4 on factor 1 (0.432) and on factor 2 (0.411) (for the other aims, we retained item 4, to have comparable results with the previous study). After removal of the cross-loading item, the exploratory factor analysis (EFA) was repeated and yielded a final factor structure. The two factors accounted for 51.3% of the total variance (eigenvalues >1.5), with the first explaining 36.9% and the second explaining 14.4% of the total variance.

EFA results were generally comparable to those obtained previously [14]. In the current sample, Factor 1 contained six inattention items, and Factor 2 contained six hyperactivity/impulsivity items. Items of the first factor (Factor 1) all had high factor loadings (all loadings >0.578) except for item 7, which had a lower though still acceptable factor loading (0.485). All items of the second factor (Factor 2) had high factor loadings (all loadings >0.503). Items on Factor 1 had low loadings on Factor 2 (all loadings <0.337) and the same was the case regarding Factor 2 item loadings on Factor 1 (all loadings <0.334).

As a final step, we examined the association between scores on the previously established subscales [14] with the currently generated factors. Subscale scores and factor scores were strongly correlated (Subscale/Factor 1: *r* = 0.95, Subscale/Factor 2: *r* = 0.95, all *p*s < 0.0001). The robust relations between previously established subscale scores and current factor scores suggest that Factor 1 is associated with inattention and Factor 2 with hyperactivity/impulsivity.

### 3.3. Aim 2/Question 2: How Reliable Is the C Dog ARS across Time (40-Day Test-Retest Reliability) at the Level of the Total Score, Subscale Scores, and Individual Item Scores?

There was strong agreement across questionnaire completions of both the inattention factor (ICC = 0.860; 95% CIs = [0.805; 0.900], *p* < 0.001) and the hyperactivity/impulsivity factor (ICC = 0.881; 95% CIs = [0.834; 0.914], *p* < 0.001), indicating excellent test–retest reliability. The ADHD total score, including all items indicated excellent test–retest reliability (ICC = 0.896; 95% CIs = [0.855; 0.925], *p* < 0.001).

To examine reliability in more depth, we examined the item-level agreement within owner ratings (see Aim 4/Question 1., Table 5). The item with the lowest temporal consistency was item 13 (53.9%) (“My/this dog can not wait as it has no self-control.”).

### 3.4. Aim 2/Question 3: To what Extent Are C Dog ARS Scores Associated with Dog Age, Sex, and Training Status (External Validity)?

#### 3.4.1. Inattention

**Figure 1:**
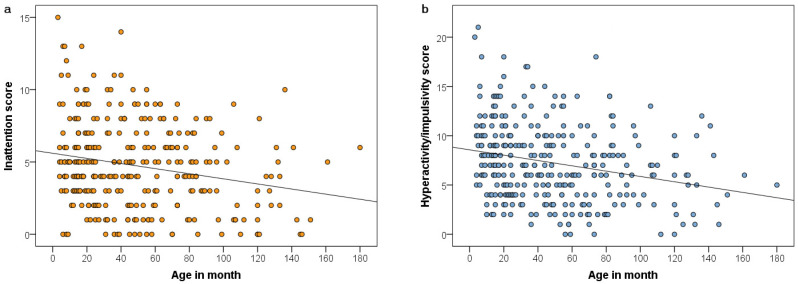
Greater age is associated with lower inattention (**a**) and with lower hyperactivity/impulsivity (**b**). The regression lines are default SPSS fitted lines representing trends in the data, with the slope corresponding to the unstandardized regression coefficient b of a linear regression equation, where Y = a (i.e., intercept) + bX (in this case, for inattention-age: y = 5.6 + −0.02 * x and y = 8.56 + −0.03 * x for hyperactivity/impulsivity-age). *Note.* The coloured circles represent subjects.

**Figure 2:**
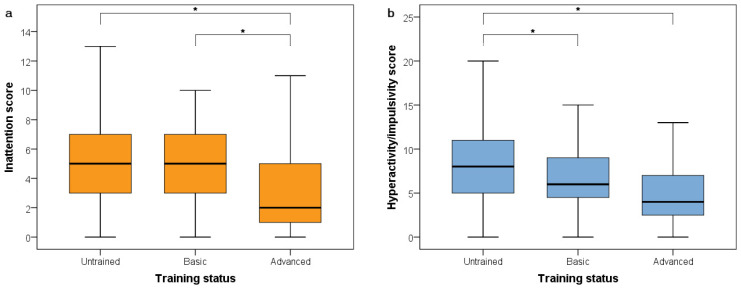
The association between training status and inattention (**a**) and hyperactivity/impulsivity (**b**); * indicates significant differences (*p* < 0.05).

Sex and the interaction between age and training status were unrelated to inattention scores. Age was associated with inattention (χ^2^_(1)_ = 5.138, *p* = 0.023); younger dogs had higher scores (Figure 1a). Following elimination of the non-significant interaction term, training status was also associated with inattention (χ^2^_(1)_ = 12.696, *p* = 0.002), with post hoc tests indicating a difference in inattention scores between dogs with no training and advanced training (*p* < 0.001, [0.80; 3.12]) and between dogs with basic and advanced training (*p* = 0.006, [0.36; 2.83]), but no difference between dogs with no training and basic training (*p* = 0.796, [−0.70; 1.43]); see Figure 2b.

#### 3.4.2. Hyperactivity/Impulsivity

Sex and the interaction between age and training status were unrelated to hyperactivity/impulsivity scores. Age was associated with hyperactivity/impulsivity (χ^2^_(1)_ = 9.878, *p* = 0.002); younger dogs had higher scores (Figure 1b). Following elimination of the non-significant interaction term, training status was also associated with hyperactivity/impulsivity (χ^2^_(1)_ = 12.763, *p* = 0.002), with post hoc tests indicating a difference in hyperactivity/impulsivity scores between dogs with no training and advanced training (*p* = 0.001, [0.72; 3.68]) and between dogs with no training and basic training (*p* = 0.050, [0.00; 2.40]), but no difference between dogs with basic training and advanced training (*p* = 0.305, [−0.51; 2.51]); see Figure 2b.

### 3.5. Aim 3/Question 1: Are There Any Ambiguous Items on the Dog ARS IDK for Owners (Dog ARS IDK-O), as Indicated by a High Proportion of Owner “I Don’t Know” Responses?

**Figure 3:**
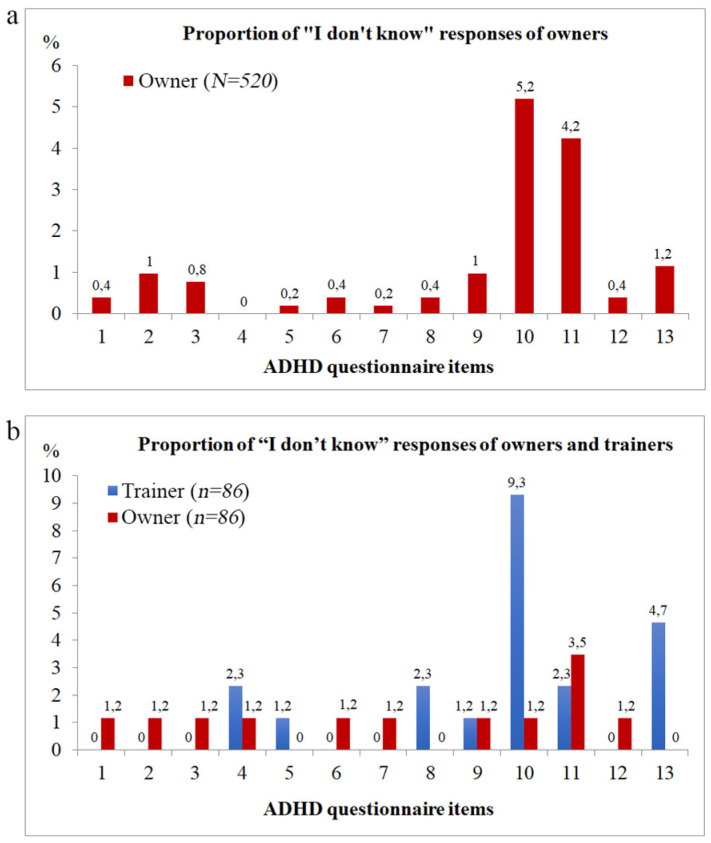
Proportion of “I don’t know” responses of (**a**) owners (*N* = 520) and (**b**) owners and trainers (*n* = 86) on the Dog ARS IDK.

The proportion of IDK responses by owners was ≤1.2% on all items, except for item 10 (“My/this dog solves simple tasks easily, but he/she often has difficulties with complicated tasks, even if those are known or have been often practiced.”) where the proportion of IDK responses were the highest (5.2%), and item 11 (“My/this dog is likely to react hastily and that is why it is failing tasks.”) with an IDK proportion of 4.2% (Figure 3a).

The average IDK response proportion for the items was 1.17%. The absolute difference between the average IDK response proportion (1.17%) and the individual item IDK response proportion was higher than 150% in the case of item 10 (344.3%) and item 11 (262.0%).

### 3.6. Aim 3/Question 2: Are There Any Ambiguous Items on the Dog ARS IDK for Trainers (Dog ARS IDK-T), as Indicated by a High Proportion of Owner “I Don’t Know” Responses?

The proportion of IDK responses by owners was ≤1.2% on all items, except for item 11 (“My/this dog is likely to react hastily and that is why it is failing tasks.”), where data indicated a higher degree of uncertainty (3.5%), similarly to the results on the larger sample of owners on 520 dogs (Figure 3a).

In the subsample of dogs where trainer ratings were available, the proportion of IDK responses by trainers were the highest in the case of item 10 (9.3%) (“My/this dog solves simple tasks easily, but he/she often has difficulties with complicated tasks, even if those are known or have been often practiced.”) and item 13 (4.7%) (“My/this dog can not wait as it has no self-control.”) (Figure 3b).

In the case of trainer responses, the average IDK response proportion for the items was 1.79%. The absolute difference between the average IDK response proportion (1.79%) and the individual item IDK response proportion was higher than 150% in the case of item 10 (420.0%) and item 13 (160.0%).

Regarding owner responses, the average IDK response proportion for the items was 1.07%. The absolute difference between the average IDK response proportion (1.07%) and the individual item IDK response proportion was higher than 150% in the case of item 11 (225.0%) (“My/this dog is likely to react hastily and that is why it is failing tasks.”).

### 3.7. Aim 4/Question 1: How Reliable Is the Dog ARS IDK across Raters (Interrater Reliability), at the Level of the Total Score, Subscale Scores, and Individual Item Scores?

ICCs indicated fair agreement between owner and trainer ratings on the inattention subscale (ICC = 0.415; 95% CIs = [0.103; 0.618], *p* < 0.001) and moderate agreement on the hyperactivity/impulsivity subscale (ICC = 0.636; 95% CIs = [0.445; 0.762], *p* < 0.001). Owner and trainer ratings exhibited moderate agreement on the ADHD total score (ICC = 0.569; 95% CIs = [0.337; 0.719], *p* < 0.001).

Interrater (owner–trainer) agreement results at the item level are summarized in Table 5. We also included here our earlier results on the item-level test–retest reliability of owner ratings for easier comparability (Aim 2/Question 2). The lowest interrater agreement was for item 10 (35.5%) (“My/this dog solves simple tasks easily, but he/she often has difficulties with complicated tasks, even if those are known or have been often practiced.”). 

## 4. Discussion

In this study, we repeated the analyses conducted by Vas and colleagues using the Dog ARS [14], addressing similar research questions and employing a similar design on new samples to examine evidence of the reliability and validity of the rating scale and to identify possible limitations. Our first aim was to examine the extent to which previous results on the Dog ARS replicate, as an index of how stable dog behaviour and/or owner rating styles are across a 14-year period (it is possible that either dog behaviour or owner rating style or both have changed since data on the Dog ARS were first collected by Vas et al. in 2007)/how stable the questionnaire is. In a similar way to human ADHD assessments, for the first time, we aimed to include expert ratings in the evaluation process, and compare expert and owner ratings. To identify possible problematic items, we included an IDK response option and examined the item level consistency in the case of within-owner and between owner–trainer agreement. We could replicate the factor structure of the Dog ARS, demonstrating evidence of good internal consistency and test–retest reliability on the inattention and hyperactivity/impulsivity subscales. Expert and owner rating comparisons revealed fair (inattention) and moderate (hyperactivity/impulsivity) agreement between raters. Based on examination of raters’ uncertainty (IDK response rates) and item level consistency, we identified three ambiguous items.

Examining whether findings replicate is important not only from the perspective of evaluating the psychometric properties of the scale but also to test its contemporary utility. Vas and colleagues conducted their study nearly fifteen years ago and, as dogs and the dog–owner relationship have likely changed a lot since then, owners may also rate their dogs’ inattention and hyperactivity/impulsivity differently. Indeed, findings of a US poll assessing changes in pet keeping habits between 2007 and 2015 indicated that pets are being increasingly viewed by owners as family members and more dogs are kept inside and allowed to sleep in their owners’ bed [51]. Moreover, in Hungary, 93.3% of dog owners consider their dogs to be family members [52].

We replicated the main findings of Vas et al. [14] in new samples, and our results confirmed the previously obtained factor structure of the questionnaire (inattention and hyperactivity/impulsivity) with a minor modification (deletion of item 4 due to cross-loading). Perhaps item 4 (“It leaves from its place when it should stay.”) reflects obedience, and thus, rather than relating only to hyperactivity/impulsivity, it is also related to attention to absolving a task and hence the cross-loading of this item. Our findings indicated good internal consistency of both subscales (inattention and hyperactivity/impulsivity). These results are inconsistent with those obtained by Lit et al., [38], who found the Dog ARS comprised three factors (inattention, hyperactivity/impulsivity-1, and hyperactivity/impulsivity-2). These differences across studies are probably due to slight differences in methodology (here, one item was removed, and a 5-point Likert-type response format scale was used instead of a 4-point scale) and differences in sample composition (a Hungarian vs. a North American dog population and/or owner bias).

Relative to item means obtained previously [14], three inattention and one hyperactivity/impulsivity item means differed in the current study. Interestingly, dogs in the present sample had higher mean scores on these items, suggesting that in the case of some traits, dogs are more inattentive or, alternatively, owners rate their dogs as more inattentive nowadays compared to 14 years ago. As cited earlier, this might be the result of the changing dog–human bond, which might shape how owners perceive their dogs’ ADHD related traits [35,51]. 

Our findings on external validity, specifically, age-related decrease in owner-rated inattention and hyperactivity/impulsivity, effect of training experience, and sex on these factors, complement earlier research. Vas and colleagues found associations between the inattention scale and age and training status, but no associations between hyperactivity/impulsivity and these variables. Some studies report a quadratic relationship between attention skills and age [53] and between activity and age [54]. In our sample, we did not observe such fine patterns.

Data on the test–retest reliability of the Dog ARS have not been published before. Our findings show both factors of the measure have good temporal consistency across 40 days. In a more in-depth examination of test–retest reliability, we also evaluated which items are ambiguous (IDK response rate) for owners and dog trainers and which items are problematic regarding consistency (percentage of agreement between two completions). For all items, test–retest findings were stronger than interrater agreement results. Regarding items 10 (“My/this dog solves simple tasks easily, but often has difficulties with complicated tasks, even those are known and have been often practiced.”), 11 (“My/this dog is likely to react hastily and that is why it is failing tasks.”), and 13 (“My/this dog cannot wait as it has no self-control.”), evaluators were uncertain and did not respond consistently, as indicated by a high IDK ratio and a low agreement between evaluations (across time in owners) and evaluators (across raters) at the item level. The uncertainty in the case of these items may stem from the fact that a question contains multiple statements, one of which may occur frequently while the other almost never. As an example, in the case of item 11, the dog may tend to react hastily, but this is not related to his performance in obedience tasks. Similarly, this problem arises in item 13: the dog may not be able to wait, but this is not related to its self-control, or waiting may mean a different context for respondents. Regarding item 10, it may be relative for each dog, which task counts as easy or difficult, and what counts as frequent exercise for each task. In a recent study where the Dog ARS’s factor structure was analysed, item 11 was excluded as it was equally loaded on the two factors, which may also indicate that the item is problematic [25]. These items could be potential targets for modification in the future, to improve the confidence and consistency of ratings and, moreover, to better reflect ADHD-like behaviours in dogs.

Owner–trainer rating comparisons revealed fair agreement between raters for the inattention scale and moderate agreement for hyperactivity/impulsivity factor. Compared to test–retest reliability results, lower owner–trainer agreement on dogs is in line with parent–teacher agreement on ratings of children [55,56,57,58]. The lower agreement on inattention and higher on the hyperactivity/impulsivity factor are also comparable to human results. Human findings report that raters find it more difficult to recognize the symptoms of inattention than detect hyperactive or impulsive symptoms of ADHD [59]. The literature on interrater reliability of assessments of internalizing and externalizing symptoms has also shown that parent–teacher agreement is higher for externalizing compared to internalizing behaviours [60]. Maybe a similar underlying mechanism could explain the difference that we found between inattention and hyperactivity/impulsivity rating comparisons.

Owners who had a trainer rating for their dog responded much more confidently (lower IDK response rates) than owners who did not have a trainer rating for the dog. It is likely that those owners who attend dog schools are generally more familiar with dog behaviours, more experienced, and thus more confident in their assessment. Owner recognition of fear-related behaviours in videos can be improved via education [61], supporting the assumption that experience on dog behaviour has an effect on ratings in general.

Compared to the earlier researcher-assisted, paper based questionnaire [14], our online data collection produced similar results, consistent with earlier findings [38]. However, we cannot determine whether the presence of the researcher can help respondents’ uncertainties regarding questionnaire items, as we do not know how much they helped the respondents in completing the questionnaire.

In sum, the Dog ARS proved to be a reliable tool to evaluate inattention and hyperactivity/impulsivity in dogs. The rating scale has a stable factor structure and good test–retest reliability, and in general, both owners and trainers can evaluate dogs with confidence. Although recent behaviour studies indicate that dogs may also show behaviours similar to human ADHD [23,62], none of the two questionnaires, which have been used so far to measure ADHD-like behaviours in dogs, provide distinction between normal and pathological levels of inattention, hyperactivity, and impulsivity. Therefore, it is still unknown whether there are dogs that can be diagnosed with ADHD applying a human-analogue methodology. Besides including questions of inattention, hyperactivity, and impulsivity characteristics, human questionnaires have a set of items which focus on functional deficits or symptom checklists used in combination with functionality assessments. This key component of ADHD has been completely ignored so far in dog ADHD-related assessments. 

Another aspect to review is the factor structure of ADHD assessment in dogs. Although many human studies measure hyperactivity/impulsivity as one dimension of ADHD, more and more evidence is being published suggesting that the factor structure of symptoms of ADHD may change during adolescence and, specifically, that hyperactivity and impulsivity symptoms may diverge at some point in adolescence [63]. Studies indicate that impulsivity diminished at a slower rate than hyperactivity during the transition to adolescence [37], and this trend in the development of these traits may explain that during adulthood a three-factor structure of ADHD (inattention, hyperactivity, and impulsivity) fits better than a two-factor structure (inattention and hyperactivity/impulsivity) applied to children [7,37,64,65,66]. Thus, it would be worth inspecting closer whether impulsivity in dogs is a distinct factor from hyperactivity [67]. In humans, the trait of impulsivity and ADHD symptom severity are important correlates of reactive/impulsive aggression [68,69], which can also be a relevant factor to investigate in dogs.

Moreover, given that the present scales were created for dog owners, there is a need to include expert ratings, such as veterinarians or dog trainers to provide a comprehensive evaluation of ADHD-like behaviours in dogs. Inclusion of expert ratings and functional impairment assessments are key components of ADHD evaluation in humans. Thus, we suggest that the present rating scale can be a basis for further development, including expert-owner ratings and functional impairment in the evaluation process, to establish whether there are diagnosable individuals with ADHD in the case of dogs.

## 5. Conclusions

Summarizing key aims and results, the current study replicated the factor structure of the Dog ARS, with evidence of good internal consistency and test–retest reliability of inattention and hyperactivity/impulsivity subscales, revealed three ambiguous items that may need modification in the future, and for the first time included expert ratings of the Dog ARS, where fair (inattention subscale) to moderate (hyperactivity/impulsivity subscale) agreement was detected between owners and trainers. Our findings add to a growing body of research by extending the dog as an animal model of human ADHD and designates potential directions for future research to increase the reliability of the ADHD measurement in dogs and its comparability with humans.

## Figures and Tables

**Table 1:** Summary of obtained factor structure and assessed psychometric properties of the Dog ARS across studies.

Study	Measure	Factors and Number of Items	Psychometric Property			
			Internal Consistency	Test–Retest Reliability	Convergent Validity	Expert Rating
Vas et al., 2007 [14]	ADHD	IA (6)	Acceptable internal consistency	–	Age is not a key correlate of ADHD	–
		H/I (7)				
Lit et al., 2010 [38]	ADHD	IA (6)	Only on a priori subscales	–	No key correlates	–
		H/I-1 (4)				
		H/I-2 (2)				
Wright et al., 2011 [15]	I	Behavioural regulation (8)	Unacceptable Cronbach’s alpha on the Responsiveness factor (=0.44)	Pearson’s correlation between test and retest scores	Two items were correlated with the total questionnaire scores, contradictory results with behaviour tests	–
		Aggression and response to novelty (5)				
		Responsiveness (5)				

Note. IA = Inattention; H = Hyperactivity; I = Impulsivity.

**Table 2:** Summary of the aims and corresponding methods and samples used in the present study.

Aim/Question	Method	Sample
A1. Replicability of the Dog ARS at the item level Q1. Stability of item ratings	Comparing current and previously obtained data: Q1. Average item ratings and SDs	Q1.: V Dog ARS (*N* = 220) vs. C Dog ARS (*N* = 319)
A2. General replicability of the Dog ARS Q1. Stability of factor structure Q2. Test-retest reliability of the Dog ARS Q3. Effect of age, sex, and training status on subscales	Comparing current and previously obtained data: Q1. Exploratory factor analysis, Cronbach’s alpha Q2. Intraclass correlation coefficient and item level, agreement rate (%) between test–retest ratings Q3. GLM	Q1. and Q3.: V Dog ARS (*N* = 220) vs. C Dog ARS (*N* = 319) Q2.: C Dog ARS vs. its retest (*n* = 140/319)
A3. Identification of ambiguous items in the Dog ARS Q1. Identify ambiguous items for owners Q2. Identify ambiguous items for trainers and owners	Q1. & Q2.: Inclusion of an “I don’t know” response option for each item	Q1.: Dog ARS IDK-O (*N* = 520) Q2.: Dog ARS IDK-T subsample: (*n* = 86/520)
A4. Owner vs. Trainer ratings on the Dog ARS Q1. Interrater reliability at the level of the total score, subscale scores, and individual item scores	Comparing owner and trainer ratings: Q1.: Intraclass correlation coefficient and item level agreement rate (%) between trainer-owner ratings	Q1.: Dog ARS IDK-T subsample: (*n* = 86/520)

Note. Abbreviations: A: aim, Q: question, V Dog ARS: previously obtained data by Vas et al. [14], C Dog ARS: current dataset on Dog ARS, GLM: Generalized Linear Models, Dog ARS IDK-O: current dataset on Dog ARS with “I don’t know” option for owners, Dog ARS IDK-T: current dataset on Dog ARS with “I don’t know” option for trainers.

**Table 3:** Descriptive statistics, cross-study differences, and indices of reliability across individual items of the V Dog ARS for the prior Vas et al. (V) (*N* = 220) [14] and the current C Dog ARS (C) sample (*N* = 319) for Aim 1/Question 1 and Aim 2/Question 1.

Items	M (*SD*)		Difference between Variance and Means (Levene’s Test and Independent Samples *t*-Tests)	Corrected Item—Total Correlation		Cronbach’s Alpha If Item Deleted		Cronbach’s Alpha	
	V	C		V	C	V	C	V	C
	Inattention subscale							0.78	0.81
1. My/this dog has learning difficulties, because it is careless or because other things easily attract its attention.	0.71 (0.73)	0.84 (0.69)	F(454) = 6.26, *p* = 0.013 * t(454) = −2.04, *p* = 0.042 *	0.58	0.72	0.74	0.75		
2. It is easy to attract my/this dog’s attention, but it also quickly loses interest.	0.82 (0.84)	0.98 (0.76)	F(441) = 13.44, *p* < 0.001 * t(441) = −2.23, *p* = 0.026 *	0.64	0.60	0.72	0.78		
3. It is difficult for my/this dog to concentrate on a task or play.	0.50 (0.69)	0.59 (0.63)	F(537) = 1.37, *p* = 0.242 *t*(537) = −1.69, *p* = 0.091	0.58	0.62	0.74	0.78		
7. It seems that my/this dog does not listen even if it knows that someone is directly speaking to him/her.	0.50 (0.70)	0.56 (0.67)	F(537) = 0.20, *p* = 0.654 *t*(537) = −0.94, *p* = 0.346	0.41	0.45	0.78	0.81		
10. My/this dog solves simple tasks easily, but he/she often has difficulties with complicated tasks, even if those are known or have been often practiced.	0.66 (0.77)	0.62 (0.67)	F(425) = 5.24, *p* = 0.022 * t(425) = 0.55, *p* = 0.584	0.48	0.47	0.76	0.81		
12. My/this dog’s attention can be easily distracted.	0.96 (0.79)	1.15 (0.72)	F(537) = 0.37, *p* = 0.545 *t*(537) = −2.84, *p* = 0.005 *	0.50	0.62	0.76	0.77		
	Hyperactivity/impulsivity subscale							0.73	0.78
4. My/this dog leaves from its place when it should stay.	1.20 (0.96)	1.08 (0.74)	F(391 = 34.60, *p* < 0.001 * t(391) = 1.58, *p* = 0.114	0.35	0.43	0.72	0.77		
5. My/this dog cannot be quiet or easily calmed.	0.81 (0.98)	0.83 (0.91)	F(537) = 2.34, *p* = 0.127 *t*(537) = −0.25, *p* = 0.806	0.40	0.42	0.71	0.77		
6. My/this dog fidgets all the time.	1.39 (1.10)	1.18 (0.93)	F(415) = 22.99, *p* < 0.001 * t(415) = 2.38, *p* = 0.018 *	0.58	0.58	0.67	0.74		
8. My/this dog is excessive or difficult to control, or if it lunges, it is difficult to hold back.	0.71 (0.81)	0.76 (0.80)	F(537) = 0.001, *p* = 0.972 *t*(537) = −0.59, *p* = 0.553	0.55	0.61	0.68	0.73		
9. My/this dog would always play and run.	1.40 (1.00)	1.30 (0.92)	F(537) = 3.54, *p* = 0.060 *t*(537) = 1.23, *p* = 0.220	0.45	0.50	0.70	0.76		
11. My/this dog is likely to react hastily, and that is why it is failing tasks.	0.93 (0.84)	1.02 (0.74)	F(537) = 7.94, *p* = 0.005 * t(537) = −1.27, *p* = 0.206	0.37	0.49	0.72	0.76		
13. My/this dog cannot wait as it has no self-control.	0.98 (0.98)	1.10 (0.90)	F(537) = 1.48, *p* = 0.224 *t*(537) = −1.51, *p* = 0.131	0.45	0.52	0.70	0.75		

Note. V = previously obtained data by Vas et al. [14]; (V Dog ARS); C = current data (C Dog ARS). *t*-tests indicated differences in the case of items 1, 2, 6, and 12, denoted by *.

**Table 4:** Factor loadings of Dog ARS items for the V Dog ARS [14] and the current C Dog ARS samples for Aim 2/Question 1.

Aimed to Measure	Item	Factor 1		Factor 2	
		V	C	V	C
Inattention	Item 3	**0.765**	**0.800**	−0.120	0.016
	Item 1	**0.729**	**0.781**	0.134	0.270
	Item 2	**0.795**	**0.772**	−0.043	−0.012
	Item 12	**0.584**	**0.700**	0.366	0.305
	Item 10	**0.664**	**0.578**	0.039	0.209
	Item 7	**0.512**	**0.485**	0.218	0.337
Hyperactivity/impulsivity	Item 6	−0.119	−0.026	**0.794**	**0.822**
	Item 9	−0.157	−0.010	**0.715**	**0.759**
	Item 8	0.308	0.325	**0.682**	**0.699**
	Item 11	0.207	0.299	**0.517**	**0.558**
	Item 13	0.096	0.334	**0.581**	**0.538**
	Item 5	0.091	0.167	**0.515**	**0.503**
	* Item 4	0.377	0.432	0.392	0.411

Note. V = previously obtained data by Vas et al. [14]; C = current data. Item 4 was removed after EFA due to cross-loading, denoted by *. Bold numbers: indicate which item belongs to which factor.

**Table 5:** Agreement between owner–trainer (interrater) (*n* = 86) and within-owner (test–retest) (*n* = 140) ratings (Aim 3/Question 2) at the item level.

Item	Interrater Agreement (Owner–Trainer)	Test–Retest Agreement (Owner)
Inattention subscale		
1. My/this dog has learning difficulties because it is careless or because other things easily attract its attention.	55.3%	67.4%
2. It is easy to attract my/this dog’s attention, but it also quickly loses interest.	47.1%	63.1%
3. It is difficult for my/this dog to concentrate on a task or play.	55.3%	68.1%
7. It seems that my/this dog does not listen even if it knows that someone is directly speaking to him/her.	52.9%	66.7%
10. My/this dog solves simple tasks easily, but he/she often has difficulties with complicated tasks, even if those are known or have been often practiced.	35.5%	63.8%
12. My/this dog’s attention can be easily distracted.	50.6%	61.7%
Hyperactivity/impulsivity subscale		
4. My/this dog leaves from its place when it should stay.	57.8%	67.4%
5. My/this dog cannot be quiet or easily calmed.	47.1%	61.0%
6. My/this dog fidgets all the time.	47.1%	58.9%
8. My/this dog is excessive or difficult to control, or if it lunges, it is difficult to hold back.	42.9%	64.5%
9. My/this dog would always play and run.	44.2%	65.2%
11. My/this dog is likely to react hastily and that is why it is failing tasks.	46.9%	62.4%
13. My/this dog cannot wait as it has no self-control.	47.6%	53.9%

## Data Availability

The data presented in this study are available on request from the corresponding author.

## Supplementary Materials

The following are available online at https://www.mdpi.com/article/10.3390/ani12070807/s1, Section S1: The Dog ASR questionnaire was used to assess owners’ ratings of the dogs; Section S2: The Dog ASR IDK questionnaire was used to assess owners’ (Dog ARS IDK-O) and trainers’ (Dog ARS IDK-T) ratings of dogs; Section S3: Information on sample demographics for the different samples.

## References

[1] Polanczyk G.V., Willcutt E.G., Salum G.A., Kieling C., Rohde L.A. (2014). ADHD prevalence estimates across three decades: An updated systematic review and meta-regression analysis. Int. J. Epidemiol..

[2] Matte B., Anselmi L., Salum G., Kieling C., Gonçalves H., Menezes A., Grevet E., Rohde L.A. (2015). ADHD in DSM-5: A field trial in a large, representative sample of 18- to 19-year-old adults. Psychol. Med..

[3] Fayyad J., De Graaf R., Kessler R., Alonso J., Angermeyer M., Demyttenaere K., de Girolamo G., Haro J.M., Karam E.G., Lara C. (2007). Cross-national prevalence and correlates of adult attention-deficit hyperactivity disorder. Br. J. Psychiatry.

[4] McBurnett K., Pfiffner L.J., Willcutt E., Tamm L., Lerner M., Ottolini Y.L., Furman M.B. (1999). Experimental Cross-Validation of DSM-IV Types of Attention-Deficit/Hyperactivity Disorder. J. Am. Acad. Child Adolesc. Psychiatry.

[5] Evans S.W., Owens J.S., Bunford N. (2014). Evidence-Based Psychosocial Treatments for Children and Adolescents with Attention-Deficit/Hyperactivity Disorder. J. Clin. Child Adolesc. Psychol..

[6] Karalunas S.L., Nigg J.T. (2020). Heterogeneity and Subtyping in Attention-Deficit/Hyperactivity Disorder—Considerations for Emerging Research Using Person-Centered Computational Approaches. Biol. Psychiatry.

[7] Barkley R.A., Murphy K.R. (2010). Impairment in Occupational Functioning and Adult ADHD: The Predictive Utility of Executive Function (EF) Ratings Versus EF Tests. Arch. Clin. Neuropsychol..

[8] Bunford N., Evans S.W., Wymbs F. (2015). ADHD and Emotion Dysregulation Among Children and Adolescents. Clin. Child Fam. Psychol. Rev..

[9] Gácsi M., Maros K., Sernkvist S., Faragó T., Miklosi A. (2013). Human Analogue Safe Haven Effect of the Owner: Behavioural and Heart Rate Response to Stressful Social Stimuli in Dogs. PLoS ONE.

[10] Topál J., Gergely G., Erdőhegyi A., Csibra G., Miklósi A. (2009). Differential Sensitivity to Human Communication in Dogs, Wolves, and Human Infants. Science.

[11] Miklósi A., Topál J., Csányi V. (2004). Comparative social cognition: What can dogs teach us?. Anim. Behav..

[12] Topál J., Román V., Turcsán B. (2019). The dog (*Canis familiaris*) as a translational model of autism: It is high time we move from promise to reality. Wiley Interdiscip. Rev. Cogn. Sci..

[13] Bunford N., Csibra B., Peták C., Ferdinandy B., Miklósi A., Gácsi M. (2019). Associations among behavioral inhibition and owner-rated attention, hyperactivity/impulsivity, and personality in the domestic dog (*Canis familiaris*). J. Comp. Psychol..

[14] Vas J., Topál J., Péch A., Miklósi A. (2007). Measuring attention deficit and activity in dogs: A new application and validation of a human ADHD questionnaire. Appl. Anim. Behav. Sci..

[15] Wright H.F., Mills D.S., Pollux P.M.J. (2011). Development and Validation of a Psychometric Tool ForAssessing Impulsivity in the Domestic Dog (*Canis Familiaris*). Int. J. Comp. Psychol..

[16] Wan M., Hejjas K., Ronai Z., Elek Z., Sasvari-Szekely M., Champagne F.A., Miklósi A., Kubinyi E. (2013). DRD4andTHgene polymorphisms are associated with activity, impulsivity and inattention in Siberian Husky dogs. Anim. Genet..

[17] Gerencsér L., Bunford N., Moesta A., Miklósi A. (2018). Development and validation of the Canine Reward Responsiveness Scale –Examining individual differences in reward responsiveness of the domestic dog. Sci. Rep..

[18] Puurunen J., Sulkama S., Tiira K., Araujo C., Lehtonen M., Hanhineva K., Lohi H. (2016). A non-targeted metabolite profiling pilot study suggests that tryptophan and lipid metabolisms are linked with ADHD-like behaviours in dogs. Behav. Brain Funct..

[19] Pelham J.W.E., Fabiano G.A., Massetti G. (2005). Evidence-Based Assessment of Attention Deficit Hyperactivity Disorder in Children and Adolescents. J. Clin. Child Adolesc. Psychol..

[20] Booster G.D., Dupaul G.J., Eiraldi R., Power T.J. (2010). Functional Impairments in Children With ADHD: Unique Effects of Age and Comorbid Status. J. Atten. Disord..

[21] Reyes A.D.L., Augenstein T.M., Wang M., Thomas S.A., Drabick D.A.G., Burgers D.E., Rabinowitz J. (2015). The validity of the multi-informant approach to assessing child and adolescent mental health. Psychol. Bull..

[22] DuPaul G.J., Power T.J., Anastopoulos A.D., Reid R. (1998). ADHD Rating Scale—IV: Checklists, Norms, and Clinical Interpretation.

[23] Hoppe N., Bininda-Emonds O.R.P., Gansloßer U. (2017). Correlates of Attention Deficit Hyperactivity Disorder (ADHD)-Like Behavior in Domestic Dogs: First Results from a Questionnaire-Based Study. Vet.- Med. Open J..

[24] Salonen M., Sulkama S., Mikkola S., Puurunen J., Hakanen E., Tiira K., Araujo C., Lohi H. (2020). Prevalence, comorbidity, and breed differences in canine anxiety in 13,700 Finnish pet dogs. Sci. Rep..

[25] Sulkama S., Puurunen J., Salonen M., Mikkola S., Hakanen E., Araujo C., Lohi H. (2021). Canine hyperactivity, impulsivity, and inattention share similar demographic risk factors and behavioural comorbidities with human ADHD. Transl. Psychiatry.

[26] Watson F., Packer R.M.A., Rusbridge C., Volk H.A. (2020). Behavioural changes in dogs with idiopathic epilepsy. Vet. Rec..

[27] Hejjas K., Vas J., Kubinyi E., Sasvari-Szekely M., Miklósi A., Rónai Z. (2007). Novel repeat polymorphisms of the dopaminergic neurotransmitter genes among dogs and wolves. Mamm. Genome.

[28] Hejjas K., Vas J., Topál J., Szántai E., Rónai Z., Székely A., Kubinyi E., Horvath Z., Sasvári-Székely M., Miklosi A. (2007). Association of polymorphisms in the dopamine D4 receptor gene and the activity-impulsivity endophenotype in dogs. Anim. Genet..

[29] Kubinyi E., Vas J., Hejjas K., Ronai Z., Brúder I., Turcsán B., Sasvari-Szekely M., Miklosi A. (2012). Polymorphism in the Tyrosine Hydroxylase (TH) Gene Is Associated with Activity-Impulsivity in German Shepherd Dogs. PLoS ONE.

[30] Lit L., Belanger J.M., Boehm D., Lybarger N., Haverbeke A., Diederich C., Oberbauer A.M. (2013). Characterization of a dopamine transporter polymorphism and behavior in Belgian Malinois. BMC Genet..

[31] Bunford N., Csibra B., Gácsi M. (2019). Individual Differences in Response to Ambiguous Stimuli in a Modified Go/No-Go Paradigm are Associated with Personality in Family Dogs. Sci. Rep..

[32] Mongillo P., Bono G., Regolin L., Marinelli L. (2010). Selective attention to humans in companion dogs, *Canis familiaris*. Anim. Behav..

[33] Bray E.E., Sammel M.D., Seyfarth R.M., Serpell J., Cheney D.L. (2017). Temperament and problem solving in a population of adolescent guide dogs. Anim. Cogn..

[34] Kubinyi E., Gosling S.D., Miklósi A. (2015). A comparison of rating and coding behavioural traits in dogs. Acta Biol. Hung..

[35] Wan M., Kubinyi E., Miklósi A., Champagne F. (2009). A cross-cultural comparison of reports by German Shepherd owners in Hungary and the United States of America. Appl. Anim. Behav. Sci..

[36] Mick E., Faraone S.V., Biederman J. (2004). Age-dependent expression of attention-deficit/hyperactivity disorder symptoms. Psychiatr. Clin. N. Am..

[37] Biederman J., Mick E., Faraone S.V. (2000). Age-Dependent Decline of Symptoms of Attention Deficit Hyperactivity Disorder: Impact of Remission Definition and Symptom Type. Am. J. Psychiatry.

[38] Lit L., Schweitzer J.B., Iosif A.-M., Oberbauer A.M. (2010). Owner reports of attention, activity, and impulsivity in dogs: A replication study. Behav. Brain Funct..

[39] Costello A.B., Osborne J.W. (2005). Best practices in exploratory factor analysis: Four recommendations for getting the most from your analysis. Pract. Assess. Res. Eval..

[40] Cicchetti D.V. (1994). Guidelines, Criteria, and Rules of Thumb for Evaluating Normed and Standardized Assessment Instruments in Psychology. Psychol. Assess..

[41] Vaz S., Falkmer T., Passmore A.E., Parsons R., Andreou P. (2013). The Case for Using the Repeatability Coefficient When Calculating Test–Retest Reliability. PLoS ONE.

[42] Riemer S., Mills D.S., Wright H. (2014). Impulsive for life? The nature of long-term impulsivity in domestic dogs. Anim. Cogn..

[43] Baker S.R., Gibson B.J., Sufi F., Barlow A.P.S., Robinson P.G., Robinson P.G. (2015). 9—The Dentine Hypersensitivity Experience Questionnaire (DHEQ): A Longitudinal Validation Study. Dentine Hypersensitivity.

[44] Rindskopf D., Smelser N.J., Baltes P.B. (2001). Reliability: Measurement. International Encyclopedia of the Social & Behavioral Sciences.

[45] Wright H.F., Mills D.S., Pollux P.M. (2012). Behavioural and physiological correlates of impulsivity in the domestic dog (*Canis familiaris*). Physiol. Behav..

[46] Brucks D., Soliani M., Range F., Marshall-Pescini S. (2017). Reward type and behavioural patterns predict dogs’ success in a delay of gratification paradigm. Sci. Rep..

[47] De Los Reyes A., Kazdin A.E. (2005). Informant Discrepancies in the Assessment of Childhood Psychopathology: A Critical Review, Theoretical Framework, and Recommendations for Further Study. Psychol. Bull..

[48] Narad M.E., Garner A.A., Peugh J.L., Tamm L., Antonini T.N., Kingery K.M., Simon J.O., Epstein J.N. (2015). Parent–teacher agreement on ADHD symptoms across development. Psychol. Assess..

[49] Shrout P.E., Fleiss J.L. (1979). Intraclass correlations: Uses in assessing rater reliability. Psychol. Bull..

[50] Sundvall L., Ingerslev H.J., Knudsen U.B., Kirkegaard K. (2013). Inter- and intra-observer variability of time-lapse annotations. Hum. Reprod..

[51] Rowan A., Kartal T. (2018). Dog Population & Dog Sheltering Trends in the United States of America. Animals.

[52] Kubinyi E., Turcsán B., Miklósi Á. (2009). Dog and owner demographic characteristics and dog personality trait associations. Behav. Process..

[53] Wallis L.J., Range F., Müller C.A., Serisier S., Huber L., Zsó V. (2014). Lifespan development of attentiveness in domestic dogs: Drawing parallels with humans. Front. Psychol..

[54] Marck A., Berthelot G., Foulonneau V., Marc A., Antero J., Noirez P., Bronikowski A.M., Morgan T., Garland T., Carter P.A. (2017). Age-Related Changes in Locomotor Performance Reveal a Similar Pattern for *Caenorhabditis elegans*, *Mus domesticus*, *Canis familiaris*, *Equus caballus*, *and Homo sapiens*. J. Gerontol. Ser. A.

[55] Hardy K.K., Kollins S.H., Murray D.W., Riddle M.A., Greenhill L., Cunningham C., Abikoff H.B., McCracken J.T., Vitiello B., Davies M. (2007). Factor Structure of Parent- and Teacher-Rated Attention-Deficit/Hyperactivity Disorder Symptoms in the Preschoolers with Attention-Deficit/Hyperactivity Disorder Treatment Study (PATS). J. Child Adolesc. Psychopharmacol..

[56] Abnorm J., Psychol C., Pennington B.F., Hartman C.A. (2007). Modeling Rater Disagreement for ADHD: Are Parents or Teachers Biased?. J. Abnorm. Child Psychol..

[57] Mitsis E.M., McKAY K.E., Schulz K.P., Newcorn J.H., Halperin J.M. (2000). Parent–Teacher Concordance for DSM-IV Attention-Deficit/Hyperactivity Disorder in a Clinic-Referred Sample. J. Am. Acad. Child Adolesc. Psychiatry.

[58] Wolraich M.L., Lambert E.W., Bickman L., Simmons T., Doffing M.A., Worley K.A. (2004). Assessing the Impact of Parent and Teacher Agreement on Diagnosing Attention-Deficit Hyperactivity Disorder. J. Dev. Behav. Pediatr..

[59] Weckerly J., Aarons G.A., Leslie L.K., Garland A.F., Landsverk J., Hough R.L. (2005). Attention on Inattention: The Differential Effect of Caregiver Education on Endorsement of ADHD Symptoms. J. Dev. Behav. Pediatr. JDBP.

[60] Faraone S.V., Biederman J., Milberger S. (1995). How Reliable Are Maternal Reports of Their Children’s Psychopathology? One-Year Recall of Psychiatric Diagnoses of ADHD Children. J. Am. Acad. Child Adolesc. Psychiatry.

[61] Flint H.E., Coe J.B., Pearl D.L., Serpell J.A., Niel L. (2018). Effect of training for dog fear identification on dog owner ratings of fear in familiar and unfamiliar dogs. Appl. Anim. Behav. Sci..

[62] Masson S., Gaultier E. (2018). Retrospective Study on Hypersensitivity-Hyperactivity Syndrome in Dogs: Long-Term Outcome of High Dose Fluoxetine Treatment and Proposal of a Clinical Score. Dog Behav..

[63] Evans S.W., Brady C.E., Harrison J., Bunford N., Kern L., State T., Andrews C. (2013). Measuring ADHD and ODD Symptoms and Impairment Using High School Teachers’ Ratings. J. Clin. Child Adolesc. Psychol..

[64] Caterino L.C., Gómez-Benito J., Balluerka N., Amador-Campos J.A., Stock W.A. (2009). Development and validation of a scale to assess the symptoms of attention-deficit/hyperactivity disorder in young adults. Psychol. Assess..

[65] Span S.A., Earleywine M., Strybel T.Z. (2002). Confirming the Factor Structure of Attention Deficit Hyperactivity Disorder Symptoms in Adult, Nonclinical Samples. J. Psychopathol. Behav. Assess..

[66] Glutting J.J., Youngstrom E.A., Watkins M.W. (2005). ADHD and College Students: Exploratory and Confirmatory Factor Structures with Student and Parent Data. Psychol. Assess..

[67] Martel M.M., Levinson C.A., Langer J.K., Nigg J.T. (2016). A Network Analysis of Developmental Change in ADHD Symptom Structure from Preschool to Adulthood. Clin. Psychol. Sci..

[68] Connor D.F., Chartier K., Preen E.C., Kaplan R.F. (2010). Impulsive Aggression in Attention-Deficit/Hyperactivity Disorder: Symptom Severity, Co-Morbidity, and Attention-Deficit/Hyperactivity Disorder Subtype. J. Child Adolesc. Psychopharmacol..

[69] Dowson J.H., Blackwell A.D. (2010). Impulsive aggression in adults with attention-deficit/hyperactivity disorder. Acta Psychiatr. Scand..

