# Supplementary material for: Evaluating ADHD Assessment for Dogs: A Replication Study
Source: Animals (Basel). 2022 Mar 22;12(7):807. doi: 10.3390/ani12070807 (PMC8997028; doi:10.3390/ani12070807)
Supplement: Supplementary file 1 [file animals-12-00807-s001.zip › animals-1590241-supplementary.pdf]

Supplementary material to:

Evaluating ADHD assessment for dogs: A replication study

Barbara Csibra [1,2] *, Nóra Bunford [3] and Márta Gácsi [1,4]

1. Eötvös Loránd University, Institute of Biology, Department of Ethology, Pázmány Péter sétány 1/C, Budapest, 1117 Hungary;
2. Doctoral School of Biology, Institute of Biology, ELTE Eötvös Loránd University, Pázmány Péter sétány 1/C, Budapest, 1117 Hungary
3. Developmental and Translational Neuroscience Research Group, Research Centre for Natural Sciences, Institute of Cognitive Neuroscience and Psychology, Magyar tudósok körútja 2, Budapest, 1117 Hungary;
4. MTA-ELTE Comparative Ethology Research Group, Pázmány Péter sétány 1/C, Budapest, 1117 Hungary;

* Correspondence: csibrabarbara@gmail.com

Section S1

The Dog ASR questionnaire [14] was used to assess owners' ratings of the dogs. Bulleted points indicate available categories provided for each question. Space was provided for alternative answers where necessary.

<u>General questions</u>

1. Owner's name (or unique identifier)
2. Email address. It is used only for the communication necessary for the tests and is not passed on to third parties.
3. The dog's name
4. The dog's sex
    - Male, Female, Neutered male, Neutered female
5. The dog's date of birth in YYYY.MM.DD format. If you DO NOT KNOW your dog's exact birth date, please, enter the following numbers: "1212.12.12." and go to the next question where you can enter the estimated age of the dog.
6. Your dog's estimated date of birth. If you don't know exactly when your dog was born, please estimate its age.
7. The dog's breed
8. The dog's training status
    - None, Basic, Advanced

<u>Dog ASR items</u>

How typical are the following statements of your dog?

1. My/ this dog has learning difficulties, because it is careless or because other things easily attract it's attention.
    - Never (0), Sometimes (1), Often (2), Very often (3)
2. It is easy to attract my/ this dog's attention, but it also quickly loses interest.
    - Never (0), Sometimes (1), Often (2), Very often (3)
3. It is difficult for my/ this dog to concentrate on a task or play.
    - Never (0), Sometimes (1), Often (2), Very often (3)
4. My/ this dog leaves from its place when it should stay.
    - Never (0), Sometimes (1), Often (2), Very often (3)
5. My/ this dog can not be quiet or easily calmed.
    - Never (0), Sometimes (1), Often (2), Very often (3)
6. My/ this dog fidgets all the time.
    - Never (0), Sometimes (1), Often (2), Very often (3)

7. It seems that my/ this dog does not listen even if it knows that someone is directly speaking to him/her.
    - Never (0), Sometimes (1), Often (2), Very often (3)
8. My/ this dog is excessive, difficult to control, or if it lunges, it is difficult to hold back.
    - Never (0), Sometimes (1), Often (2), Very often (3)
9. My/ this dog would always play and run.
    - Never (0), Sometimes (1), Often (2), Very often (3)
10. My/ this dog solves simple tasks easily, but he/she often has difficulties with complicated tasks, even if those are known or have been often practiced.
    - Never (0), Sometimes (1), Often (2), Very often (3)
11. My/ this dog is likely to react hastily and that is why it is failing tasks.
    - Never (0), Sometimes (1), Often (2), Very often (3)
12. My/ this dog's attention can be easily distracted.
    - Never (0), Sometimes (1), Often (2), Very often (3)
13. My/ this dog can not wait as it has no self-control.
    - Never (0), Sometimes (1), Often (2), Very often (3)

Section S2

The Dog ASR IDK questionnaire was used to assess owners' (Dog ARS IDK-O) and trainers' (Dog ARS IDK-T) ratings of dogs. The general questions for owners (Dog ARS IDK-O) and for trainers (Dog ARS IDK-T) differed, thus these questions listed separately. Bulleted points indicate available categories provided for each question. Space was provided for alternative answers where necessary.

<u>General questions (only for Dog ARS IDK-O)</u>

1. Owner's name (or unique identifier)
2. Email address. It is used only for the communication necessary for the tests and is not passed on to third parties.
3. The dog's name
4. The dog's sex
    - Male, Female, Neutered male, Neutered female
5. The dog's date of birth in YYYY.MM.DD format. If you DO NOT KNOW your dog's exact birth date, please, enter the following numbers: "1212.12.12." and go to the next question where you can enter the estimated age of the dog.
6. Your dog's estimated date of birth. If you don't know exactly when your dog was born, please estimate its age.
7. The dog's breed
8. The dog's training status
    - None, Basic, Advanced

<u>General questions (only for Dog ARS IDK-T)</u>

1. Trainer's name (or unique identifier)
2. Email address. It is used only for the communication necessary for the tests and is not passed on to third parties.
3. Owner's name
4. The dog's name

Dog ASR IDK items

How typical are the following statements of the dog?

1. My/ this dog has learning difficulties, because it is careless or because other things easily attract it's attention.
    - Never (0), Sometimes (1), Often (2), Very often (3), I don't know (-)
2. It is easy to attract my/ this dog's attention, but it also quickly loses interest.
    - Never (0), Sometimes (1), Often (2), Very often (3), I don't know (-)
3. It is difficult for my/ this dog to concentrate on a task or play.
    - Never (0), Sometimes (1), Often (2), Very often (3), I don't know (-)
4. My/ this dog leaves from its place when it should stay.
    - Never (0), Sometimes (1), Often (2), Very often (3), I don't know (-)

5. My/ this dog can not be quiet or easily calmed.
    - Never (0), Sometimes (1), Often (2), Very often (3), I don't know (-)
6. My/ this dog fidgets all the time.
    - Never (0), Sometimes (1), Often (2), Very often (3), I don't know (-)
7. It seems that my/ this dog does not listen even if it knows that someone is directly speaking to him/her.
    - Never (0), Sometimes (1), Often (2), Very often (3), I don't know (-)
8. My/ this dog is excessive, difficult to control, or if it lunges, it is difficult to hold back.
    - Never (0), Sometimes (1), Often (2), Very often (3), I don't know (-)
9. My/ this dog would always play and run.
    - Never (0), Sometimes (1), Often (2), Very often (3), I don't know (-)
10. My/ this dog solves simple tasks easily, but he/she often has difficulties with complicated tasks, even if those are known or have been often practiced.
    - Never (0), Sometimes (1), Often (2), Very often (3), I don't know (-)
11. My/ this dog is likely to react hastily and that is why it is failing tasks.
    - Never (0), Sometimes (1), Often (2), Very often (3), I don't know (-)
12. My/ this dog's attention can be easily distracted.
    - Never (0), Sometimes (1), Often (2), Very often (3), I don't know (-)
13. My/ this dog can not wait as it has no self-control.
    - Never (0), Sometimes (1), Often (2), Very often (3), I don't know (-)

Section S3

Information on sample demographics for the different samples. Sample sizes differed across research questions and were accordingly indicated separately below for each question.

1. Aim 1/ Question 1: Is there a difference between the item ratings of the current (C Dog ARS) and the prior sample (V Dog ARS)?

1. a Previously obtained sample on Dog ARS by Vas et al. (2007)

The sample and information are based on the Vas et al. 2007 publication [14], sampling was performed by Vas and colleagues. The sample consisted of 220 dogs: 106 males and 114 females (no data on neutering status, *M*age=35.56 months, *SD*=7.79).

The distribution of the breeds in the sample:

25 mixed breed dogs, 10 Collies, 10 Golden Retriever, 10 Groenendael, 10 Tervueren, 10 German Shepherds, 8 Border Collie, 7 Siberian Huskies, 6 German Boxers, 6 Hungarian Vizslas, 5 Great Dane, 5 Mudi, 5 English Pointers, 5 Samoyed, 4 American Staffordshire Bull Terrier, 4 Labrador Retriever, 4 Malinoi, 4 Moscow Watchdogs, 4 Shetland Sheepdog, 3 English Cocker Spaniel, 3 Dogo Argentino, 3 Australian Kelpies, 3 Central Asia Shepherd Dog, 3 German Pointer Dogs, 3 Parson Russell Terrier, 3 Pumi, 3 Toy Poodle, 3 West Highland White Terrier, 2 Bulldog, 2 Basset Hound, 2 Saint Bernard, 2 Bernese Mountain Dog, 2 Dalmatian, 2 Dobermann, 2 Kavkazskaya Ovcharka, 2 Rottweiler, 2 Miniature Schnauzer, 2 Small Spitz, 2 Welsh Terrier, 2 Medium Poodle, 1 Airedale Terrier, 1 American Bulldog, 1 Appenzeller Sennenhund, 1 Berger de Beauce, 1 Bichon Frise, 1 Old English Sheepdog, 1 Bullmastiff, 1 Cane Corso, 1 Cardigan Welsh Corgi, 1 Chow Chow, 1 Fox Terrier, 1 Hovawart, 1 Irish Red Setter, 1 Jack Russell Terrier, 1 Deutscher Jagdterrier, 1 American White Shepherd Dog, 1 Lhasa Apso, 1 Maltese, 1 Miniature Pinscher, 1 Puli, 1 Rhodesian Ridgeback, 1 Standard Schnauzer, 1 Giant Schnauzer, 1 Miniature Spitz, 1 Dachshund, 1 Whippet, 1 Yorkshire Terrier.

1. b Current sample on Dog ARS

The present sample consisted of 319 dogs: 162 male (83 intact males, 79 neutered males) and 157 female (61 intact females, 96 spayed females) dogs (*M*age=48.44 months, *SD*=36.23).

The distribution of the breeds in the sample:

88 mixed breed dogs, 1 Akita Inu, 1 American Pitbull Terrier, 1 American Cocker Spaniel, 5 American Staffordshire Terrier, 23 English Cocker Spaniel, 3 English Pointers, 1 Australian Cattle Dog, 3 Australian Shepherds, 2 Beagle, 1 Bedlington Terrier, 1 Groenendael, 3 Malinois, 1 Tervuren, 2 Bernese

Mountain Dog, 13 Border Collie, 1 Border Terrier, 2 Boston Terrier, 1 Briard, 5 Bullterrier, 2 Cane Corso, 3 Cavalier King Charles Spaniel, 2 Czechoslovakian Wolfdogs, 5 Chihuahua, 1 Dobermann, 1 Fox Terrier (Smooth-haired), 1 Fox Terrier (Wire-haired), 2 French Bulldogs, 9 Golden Retriever, 2 Havaneses, 1 Hovawart, 5 Irish Setters, 2 Jack Russell Terrier, 1 East-European Shepherd, 2 Standard Schnauzer, 2 Kuvasz, 14 Labrador Retriever, 1 Lagotto Romagnolo, 2 Landseer, 3 Hungarian Greyhounds, 10 Vizsla, 1 Maltese, 1 Pug, 5 Mudi, 21 German Shepherds, 1 German Pinscher, 2 German Pointers (Short-haired), 4 Giant Schnauzer, 1 Black Russian Terrier, 1 Papillon, 1 Pomeranian, 3 Puli, 1 Pumi, 4 Rottweiler, 1 Yugoslavian Shepherd Dog, 1 Shiba Inu, 2 Shih-tzu, 2 Collie (Rough), 2 Staffordshire Bull Terrier, 5 White Swiss Shepherd Dog, 1 Siberian Husky, 1 Dachshund (Wire-haired), 1 Dachshund (Long-haired), 4 Dachshund (Short-haired), 1 Miniature Dachshund (Short-haired), 1 Miniature Dachshund (Long-haired), 1 Tibetan Terrier, 2 Miniature Pinschers, 3 Miniature Schnauzers, 1 Medium Poodle, 1 Toy Poodle, 2 Standard Poodle, 1 Volpino Italiano, 6 Whippet, 5 Yorkshire Terrier.

2. Aim 2/ Question 1: Is there a difference between the factor structure of the current (C Dog ARS) and the prior sample (V Dog ARS)?

For this research question, the same sample was used as in Aim1/ Question1.

3. Aim 2/ Question 2: How reliable is the C Dog ARS across time (40-day test-retest reliability) at the level of the total score, subscale scores, and individual item scores?

This sample is a subsample of the Dog ARS replication sample (*n*=140/319, see Aim 1/ Question 1). We included those dogs, where the owners filled out the Dog ARS again within a short time interval, for test-retest analysis.

The sample consisted of 140 dogs: 74 male (37 intact males, 37 neutered males) and 66 female (27 intact females, 39 spayed females) dogs (*M*age=48.65 months, *SD*=36.58).

The distribution of the breeds in the sample:

39 mixed breed dogs, 1 Akita Inu, 1 American Pitbull Terrier, 3 American Staffordshire Terrier, 9 English Cocker Spaniel, 2 English Pointers, 1 Australian Shepherd, 2 Beagle, 1 Groenendael, 1 Malinois, 1 Bernese Mountain Dog, 7 Border Collie, 2 Bullterrier, 1 Cane Corso, 1 Cavalier King Charles Spaniel, 1 Czechoslovakian Wolfdog, 2 Chihuahua, 1 Dobermann, 1 East-European Shepherd, 6 Golden Retriever, 1 Havanese, 3 Irish Setters, 2 Jack Russell Terrier, 2 Kuvasz, 4 Labrador Retriever, 1 Lagotto Romagnolo, 2 Landseer, 3 Hungarian Greyhound, 5 Vizsla, 1 Pug, 2 Mudi, 6 German Shepherds, 1 German Pointer (Short-haired), 2 Giant Schnauzer, 1 Papillon, 1 Puli, 2 Rottweiler, 1 Yugoslavian Shepherd Dog, 1 Shiba Inu, 1 White Swiss Shepherd Dog, 3 Dachshund (Short-haired), 1 Miniature Dachshund (Short-haired), 1 Miniature Dachshund (Long-haired), 1 Standard Poodle, 3 Whippet, 3 Yorkshire Terrier.

4. Aim 2/ Question 3: To what extent are C Dog ARS scores associated with dog age, sex and training status (external validity)?

For this research question, the same sample was used as in Aim1/ Question1.

5. Aim 3/ Question 1: Are there any ambiguous items on the Dog ARS IDK-O, as indicated by a high proportion of owner "I don't know" responses?

The sample consisted of 520 dogs: 227 male (120 intact males, 107 neutered males) and 293 female (98 intact females, 195 spayed females) dogs (*M*age=55.01 months, *SD*=39.59).

The distribution of the breeds in the sample:

185 mixed breed dogs, 8 Beagle, 6 Bischon Havanese, 4 Greyhound, 4 Airedale Terrier, 2 Alaskan Malamutes, 2 American Pitbull Terrier, 4 American Staffordshire Terrier, 4 Bullterrier, 8 English Cocker Spaniel, 4 Argentin Dogs, 2 Australian Kelpies, 7 Malinois, 4 Groenendael, 2 Berger De Brie, 4 Bernese Mountain Dog, 16 Border Collie, 6 Boston Terrier, 9 Boxers, 2 Cairn Terrier, 7 Cane Corso, 2 Chow Chow, 4 Collie (Rough), 4 Chihuahua, 4 Dobermann, 2 Transylvanian Hounds, 2 Fox Terrier (Smooth-haired), 12 Golden Retriever, 2 Gordon Setter, 2 Dutch Shepherd Dogs (Short-haired), 5 Siberian Huskies, 3 Hovawart, 1 Irish Setter, 5 Jack Russell Terrier, 1 Canary Mastiff, 2 Komondor, 4 Kuvasz, 2 Labradoodles, 20 Labrador Retriever, 2 Lagotto Romagnolo, 8 Hungarian Greyhound, 16 Vizsla, 1

Manchester Terrier, 3 Maltese, 1 Mudi, 2 Pug, 1 Moscow Watchdog, 1 Great Dane, 22 German Shepherds, 2 German Pointers (Short-haired), 2 Standard Poodle, 10 Toy Poodle, 1 Medium Poodle, 2 Black Russian Terrier, 6 Pekingese, 5 Papillon, 1 Parson Russel Terrier, 1 Great Pyreneeses, 1 Miniature Schnauzer, 2 Puli, 3 Rottweiler, 3 Samoyed, 3 Shetland Sheepdog, 4 Shiba Inu, 6 Shih-tzu, 2 Miniature Spitz, 7 White Swiss Shepherd Dog, 3 Miniature Dachshund (Wire-haired), 4 Dachshund (Short-haired), 2 Tibetan Terriers, 2 Tibetan Mastiffs, 4 Miniature Pincher, 1 Miniature Dachshund (Short-haired), 7 West Highland White Terrier, 2 Whippets, 9 Yorkshire Terrier.

6. Aim 3/ Question 2: Are there any ambiguous items on the Dog ARS IDK-T, as indicated by a high proportion of trainer"I don't know" responses?

The sample consisted of 86 dogs: 40 male (19 intact males, 21 neutered males) and 46 female (13 intact females, 33 spayed females) dogs (*M*age=44.31 months, *SD*=28.96).

The distribution of the breeds in the sample:

26 mixed breed dogs, 1 Airedale Terrier, 5 English Cocker Spaniel, 1 Australian Kelpie, 2 Malinois, 9 Border Collie, 1 Boston Terrier, 2 Boxer, 1 Cairn Terrier, 3 Golden Retriever, 1 Groenendael, 1 Labradoodle, 7 Labrador Retriever, 1 Lagotto Romagnolo, 2 Hungarian Greyhounds, 3 Vizsla, 1 Manchester Terrier, 2 German Shepherds, 1 Parson Russel Terrier, 1 Pumi, 1 Rottweiler, 2 Sheltie, 1 Shiba Inu, 1 Spitz, 1 White Swiss Shepherd Dog, 1 Dachshund (Short-haired), 1 Kaninchen Dachshund, 1 Tibetan Terrier, 1 Toy Poodle, 4 Standard Poodle, 1 Newfoundland.

7. Aim 4/ Question 1: How reliable is the Dog ARS across raters (interrater reliability), at the level of the total score, subscale scores, and individual item scores?

For this research question, the same sample was used as in Aim3/ Question2.
